# Folate receptor-targeted positron emission tomography of experimental autoimmune encephalomyelitis in rats

**DOI:** 10.1186/s12974-019-1612-3

**Published:** 2019-12-03

**Authors:** Petri Elo, Xiang-Guo Li, Heidi Liljenbäck, Semi Helin, Jarmo Teuho, Kalle Koskensalo, Virva Saunavaara, Päivi Marjamäki, Vesa Oikonen, Jenni Virta, Qingshou Chen, Philip S. Low, Juhani Knuuti, Sirpa Jalkanen, Laura Airas, Anne Roivainen

**Affiliations:** 1 Turku PET Centre, University of Turku, Turku, Finland; 2 Turku PET Centre, Åbo Akademi University, Turku, Finland; 3 Turku Center for Disease Modeling, University of Turku, Turku, Finland; 4 Turku PET Centre, Turku University Hospital, Turku, Finland; 5 Department of Medical Physics, Turku University Hospital, Turku, Finland; 6 Department of Chemistry, Purdue University, West Lafayette, IN USA; 7 MediCity Research Laboratory, University of Turku, Turku, Finland; 8 Department of Neurology, Turku University Hospital, Turku, Finland

**Keywords:** Folate receptor, Experimental autoimmune encephalomyelitis, Inflammation, Positron emission tomography, Macrophages

## Abstract

**Background:**

Folate receptor-β (FR-β) is a cell surface receptor that is significantly upregulated on activated macrophages during inflammation and provides a potential target for folate-based therapeutic and diagnostic agents. FR-β expression in central nervous system inflammation remains relatively unexplored. Therefore, we used focally induced acute and chronic phases of experimental autoimmune encephalomyelitis (EAE) to study patterns of FR-β expression and evaluated its potential as an in vivo imaging target.

**Methods:**

Focal EAE was induced in rats using heat-killed Bacillus Calmette-Guérin followed by activation with complete Freund’s adjuvant supplemented with *Mycobacterium tuberculosis*. The rats were assessed with magnetic resonance imaging and positron emission tomography/computed tomography (PET/CT) at acute (14 days) and chronic (90 days) phases of inflammation. The animals were finally sacrificed for ex vivo autoradiography of their brains. PET studies were performed using FR-β-targeting aluminum [^18^F]fluoride-labeled 1,4,7-triazacyclononane-*1,4,7*-triacetic acid conjugated folate ([^18^F]AlF-NOTA-folate, ^18^F-FOL) and 18 kDa translocator protein (TSPO)-targeting *N*-acetyl-*N*-(2-[^11^C]methoxybenzyl)-2-phenoxy-5-pyridinamine (^11^C-PBR28). Post-mortem immunohistochemistry was performed using anti-FR-β, anti-cluster of differentiation 68 (anti-CD68), anti-inducible nitric oxide synthase (anti-iNOS), and anti-mannose receptor C-type 1 (anti-MRC-1) antibodies. The specificity of ^18^F-FOL binding was verified using in vitro brain sections with folate glucosamine used as a blocking agent.

**Results:**

Immunohistochemical evaluation of focal EAE lesions demonstrated anti-FR-β positive cells at the lesion border in both acute and chronic phases of inflammation. We found that anti-FR-β correlated with anti-CD68 and anti-MRC-1 immunohistochemistry; for MRC-1, the correlation was most prominent in the chronic phase of inflammation. Both ^18^F-FOL and ^11^C-PBR28 radiotracers bound to the EAE lesions. Autoradiography studies verified that this binding took place in areas of anti-FR-β positivity. A blocking assay using folate glucosamine further verified the tracer’s specificity. In the chronic phase of EAE, the lesion-to-background ratio of ^18^F-FOL was significantly higher than that of ^11^C-PBR28 (*P* = 0.016).

**Conclusion:**

Our EAE results imply that FR-β may be a useful target for in vivo imaging of multiple sclerosis-related immunopathology. FR-β-targeted PET imaging with ^18^F-FOL may facilitate the monitoring of lesion development and complement the information obtained from TSPO imaging by bringing more specificity to the PET imaging armamentarium for neuroinflammation.

## Background

Multiple sclerosis (MS) is an autoimmune disease of the central nervous system (CNS) that leads to demyelination and axonal damage [1]. In the early stages of MS, during the relapsing-remitting disease (RRMS), CNS pathology is confined to focal inflammatory lesions in the white matter and occasionally in normal-appearing white matter (NAWM) and gray matter [1–5]. With the progression of the disease into the secondary progressive phase (SPMS), the pathology is altered and is characterized by microglial activation and widespread damage in areas of cortical gray matter, white matter, and NAWM [6–8]. In this secondary phase, the disease is no longer treatable using the anti-inflammatory treatments that are effective during the RRMS phase [6]. In SPMS, microglia have also been considered essential for driving both demyelination and remyelination [6, 9].

MS is a human disease, and experimental autoimmune encephalomyelitis (EAE) is the most widely used animal model for MS. EAE has provided vast amounts of information on the inflammatory components of MS, and some of the currently available treatments have been developed using EAE [10, 11]. Conventional EAE models typically exhibit lesions that are disseminated throughout the whole CNS and spinal cord, and that are difficult to monitor, quantify, and measure [10], while the clinically more relevant focal delayed-type hypersensitivity experimental autoimmune encephalomyelitis (*f*DTH-EAE) can be used in experiments comparing lesion size, progression, and inflammatory activity [12]. DTH lesions closely mimic those observed in MS with respect to the breakdown of the blood-brain barrier (BBB) in the acute inflammatory phase, demyelination, microglial activation, and macrophage recruitment [12]. In addition, DTH lesions resemble those occurring in the progressive phase of MS when they advance to the chronic phase of inflammation.

Most of the positron emission tomography (PET) ligands used for imaging of neuroinflammation target the 18 kDa translocator protein (TSPO) [13]. TSPO has been shown to be upregulated in activated macrophages, astrocytes, and microglia during inflammation and brain injury, and is hence considered to be a marker of inflammation in the brain [14, 15]. Use of the second-generation TSPO-targeting PET ligand *N*-acetyl-*N*-(2-[^11^C]methoxybenzyl)-2-phenoxy-5-pyridinamine (^11^C-PBR28) has become a well-established agent for imaging neuroinflammatory conditions in animal models and patients with MS [16]. Although ^11^C-PBR28 has overcome some of the limitations of traditional TSPO-targeted PET imaging agents, such as those associated with (*R*)-[^11^C]PK11195 [17], it is still subject to certain restrictions, such as a relatively low receptor affinity and a limited capacity to measure subtle in vivo TSPO expression in the brain during inflammation, which thereby prevents its use in routine clinical practice [17, 18]. Moreover, imaging of TSPO binding is not considered to be microglia-specific, and TSPO imaging lacks the ability to capture the heterogeneity and highly dynamic macrophage/microglia activation patterns [19]. Thus, a new macrophage/microglia-targeting PET radioligand showing a better signal-to-background ratio and demonstrating the possibility to visualize subsets of microglia and macrophage cells would constitute a welcomed approach for PET imaging of MS, as microglial activation affects the progression of the disease and lesion load [20].

Healthy cells acquire their folate (folic acid) using reduced folate carriers and/or the proton-coupled folate transporter, which are needed for normal cell survival and proliferation [21, 22]. However, during inflammation, folate uptake by activated macrophages is mediated primarily by the beta isoform of the folate receptor (FR-β) that exhibits ~ 1000 higher affinity for folate than the reduced folate carrier. Because FR-β is not expressed on resting macrophages or any other cell type, it constitutes an excellent marker for inflammatory conditions such as rheumatoid arthritis, Crohn’s disease, and atherosclerosis [22, 23]. Therefore, it has been a target for the development of folate-based imaging agents for conditions overexpressing FR [22]. Aluminum [^18^F]fluoride-labeled 1,4,7-triazacyclononane-*1,4,7*-triacetic acid conjugated folate ([^18^F]AlF-NOTA-folate, ^18^F-FOL) has recently been studied as a PET imaging agent for targeting FRs in tumor xenografts [24] and inflammatory atherosclerotic lesions [25]. However, it still remains to be determined whether ^18^F-FOL can target FR-β in rats with chronic *f*DTH-EAE lesions where the BBB has been restored after the acute inflammatory phase.

The current knowledge of FR-β in CNS inflammation is very limited [22, 23]. As macrophages and microglia are believed to be relevant in the pathogenesis of MS and EAE [7, 8, 12], it could be expected that FR-β overexpression is involved in the pathology of these diseases. Several studies highlight the growing evidence of functional heterogeneity in macrophage and microglia phenotypes during chronic inflammatory reactions in MS and EAE [1, 12, 19]. For example, an imbalance towards iNOS-positive has been shown to promote inflammation in relapsing EAE, whereas an equilibrium of iNOS/Arg-1-positive cells is indicative of milder EAE and spontaneous recovery. However, the pro-inflammatory bias of microglia expression in individual rat models of EAE appears high [26]. In addition, there is a lack of understanding of the different functional phenotypes in MS, as microglia and macrophages have shown intermediate phenotypes, and the polarization patterns in different stages of lesion development are unclear, which could derive from the absence of unique markers defining these functional phenotypes [27]. However, CD206 (mannose receptor)-positive microglia are known to have an essential role in successful remyelination during the active inflammatory phase [28]. Therefore, FR-β expression patterns in macrophages and microglia during their polarization to pro-inflammatory or anti-inflammatory subtypes in chronic focal EAE, patterns that mimic progressive MS, may provide new details on the regulatory processes, iNOS/MRC-1 ratio imbalances, and repair mechanisms occurring in EAE.

In this study, we investigated the application of ^18^F-FOL for evaluation and monitoring of the progression of neuroinflammatory lesions in a rat model of MS and endeavored to determine whether FR-β expression correlates with disease progression and neuroinflammation during the time course of DTH lesions in focal EAE. In addition, we studied whether FR-β expression is indicative of either the iNOS-positive or MRC-1-positive phenotypes of microglia and macrophage in focal EAE, and whether they could contribute to inflammation severity, and possibly also to recovery from the active inflammatory phase. In vivo PET imaging was performed with the folate-based radiotracer ^18^F-FOL to assess its accumulation in inflammatory lesions. The in vivo PET data were supported with ex vivo autoradiography measurements. Histology and immunohistochemistry were used to clarify the relationship between ^18^F-FOL uptake, FR-β expression, and activation patterns of iNOS-positive and MRC-1-positive macrophages and microglia during disease development. For comparison purposes, we used the second-generation TSPO-targeting imaging agent ^11^C-PBR28 as a baseline control for FR-β-targeted PET imaging with ^18^F-FOL.

## Methods

### Induction of the disease model

Twenty rats (*n* = 20) underwent stereotactic surgery. The rats were first anesthetized using a mixture of 4–5% isoflurane (Piramal Healthcare, Northumberland, UK) and oxygen (500–700 mL/min), and a subcutaneous (s.c.) injection of 100 μL (0.05 mg/kg) of buprenorphine (Temgesic, Indivior, Berkshire, UK). Anesthesia was maintained with 2–2.5% isoflurane (400–500 mL/min), and body temperature was maintained using a heating blanket. After setting the rat in a stereotactic frame, a short incision was made to the scalp to expose the skull, and a 1.0 mm diameter hole was drilled 1.0 mm anterior and 3.0 mm lateral from the bregma at the depth of 5.0 mm from the surface of the cortex. Two microliters of heat-killed Bacillus Calmette-Guérin (BCG, a kind gift from Professor Daniel Anthony, Department of Pharmacology, University of Oxford, UK) suspension (5 × 10^5^ organisms in 1 μL of saline) was then injected using a Hamilton micro-syringe (Hamilton Bonaduz AG, Bonaduz, Switzerland).

### Peripheral lesion activation

Four weeks after the intracerebral BCG injection, the active inflammatory response in the CNS was initiated by an intradermal injection of 1.5 mg of *Mycobacterium tuberculosis* (TB; heat-killed *Mycobacterium tuberculosis*-H37Ra, InvivoGen, San Diego, CA, USA) in a complete Freund’s adjuvant (CFA)/saline emulsion (100 μL, Sigma Aldrich, St. Louis, MO, USA). Prior to the procedure, the rats were anesthetized as described above. After the operation, the rats were allowed to lie on the heating pad to recover from the anesthesia. This sensitization procedure induces an immune cell-mediated response against the CNS lesion, resulting in the development of focal DTH-type chronic lesions with microglial activation and macrophage recruitment [29].

### Animals and experimental design

Adult male Lewis rats (3–4 months, *n* = 20, 235 ± 9 g) were obtained from Charles River (Sulzfeld, Germany). They were allowed to acclimatize to the animal housing environment for 1 week prior to any experimental procedures, with food and tap water being available ad libitum for all rats*.*

The rats were randomly divided into two groups, and those in group A (*n* = 10) underwent 3-T magnetic resonance imaging (MRI) with a gadolinium (Gd)-based contrast agent and a rat-dedicated brain coil at 13 days post-lesion activation, to evaluate the lesion characteristics and BBB status. PET/computed tomography (CT) was performed with ^18^F-FOL (*n* = 5) or ^11^C-PBR28 (*n* = 5) at 14 days post-lesion activation to assess brain levels of FR-β and TSPO, respectively. After in vivo PET/CT imaging, the rats were sacrificed for ex vivo biodistribution analysis, and for autoradiographical, histological, and immunohistochemical analysis of brain sections.

**Fig. 1:**
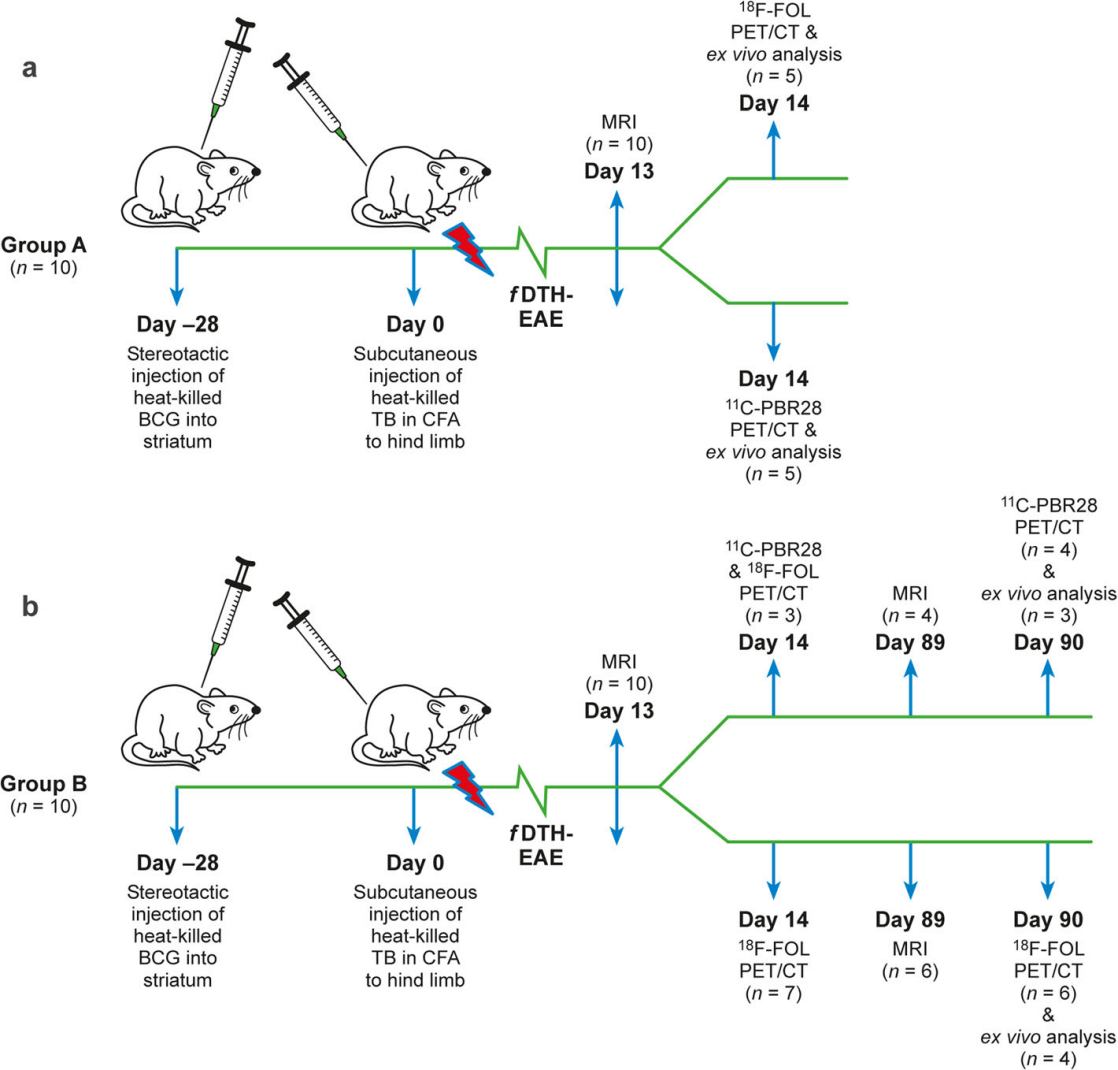
Experimental study design for acute and chronic *f*DTH-EAE models. **a** Rats in group A were studied with magnetic resonance imaging (MRI) on day 13 and with ^18^F-FOL or ^11^C-PBR28 positron emission tomography/computed tomography (PET/CT) on day 14 followed by ex vivo analyses. **b** Rats in group B were studied in both acute and chronic phases with MRI, ^18^F-FOL, or ^11^C-PBR28 PET/CT, plus ex vivo analyses at the end of the study. BCG = Bacillus Calmette-Guérin, TB = *Mycobacterium tuberculosis*, CFA = complete Freund’s adjuvant

All rats in group B (*n* = 10) were examined with MRI and ^18^F-FOL PET/CT at 13 and 14 days post-lesion activation, respectively. In addition, a subgroup of rats also underwent PET/CT imaging with ^11^C-PBR28 (*n* = 3) 4 h prior to the ^18^F-FOL PET/CT. The rats were re-evaluated with MRI and PET/CT at 89 and 90 days, respectively, post-lesion activation, using ^18^F-FOL (*n* = 6) or ^11^C-PBR28 (*n* = 4). After the last PET/CT imaging, the rats were sacrificed and examined as described above for group A (Fig. 1). Immunohistochemical staining was performed with anti-FR-β, anti-CD68, anti-iNOS, and anti-MRC-1 antibodies, to evaluate the phenotypic characteristics of the inflammatory cells during the acute and chronic phases of *f*DTH-EAE lesions (Table 1).

**Table 1:** Characteristics of the studied animals

	Group A	Group B	
	*f*DTH-EAE Days 13/14	*f*DTH-EAE Days 13/14	*f*DTH-EAE Days 89/90
Animals (total *n*)	10	10	
Weight (g)	431 ± 24	455 ± 20	502 ± 49
Lesion area (mm^2^)	0.061 ± 0.027	ND	1.3 ± 0.51
^18^F-FOL			
PET/CT (*n*)	5	10	6
Brain autoradiography (*n*)	5	ND	4
^11^C-PBR28			
PET/CT (*n*)	5	3	4
Brain autoradiography (*n*)	5	ND	3
MRI	10	10	10
Histology/immunohistochemistry			
H&E (*n*)	10	ND	9
Anti-FR-β (*n*)	10	ND	8
Anti-CD68 (*n*)	10	ND	8
Anti-iNOS (*n*)	10	ND	8
Anti-MRC-1 (*n*)	9	ND	8
LFB (*n*)	10	ND	9

*ND* not determined

In addition, 12 healthy Lewis rats were used for evaluation of in vivo stability of ^18^F-FOL and the brain of one healthy Lewis rat was examined by anti-FR-β immunohistochemical staining.

All animal experiments were approved by the national Animal Experiment Board of Finland and the Regional State Administrative Agency for Southern Finland (permission number: ESAVI/3046/04.10.07/2014) and were conducted in accordance with the relevant European Union directive.

### MRI

MRI was performed for rats in group A on day 13 after disease activation (*n* = 10) and for rats in group B on both days 13 (*n* = 10) and 89 (*n* = 6) after lesion activation. MRI was acquired using a clinical Philips Achieva 3 T device (Philips Health Care, Amsterdam, The Netherlands). Animals were first anesthetized on the heating pad with 4–5% isoflurane and oxygen (500–700 mL/min), with the anesthesia then being lowered to maintenance levels of 2–2.5% isoflurane (400–500 mL/min). A cannula was placed into the tail vein for the injection of 100 μL of the Gd-contrast agent (DOTAREM 279.3 mg/mL, Guerbet, Roissy, France) 10 min before acquiring post-contrast T1-weighted MRI to ascertain BBB integrity. For MRI, the rats were set in a rat-dedicated brain coil (Rat Brain Array 4, RAPID Biomedical GmbH, Rimpar, Germany). During imaging, maintenance levels of 2–2.5% isoflurane (400–500 mL/min) preserved the anesthesia, and an external heating system (RAPID Air Heating Control, RAPID Biomedical GmbH, Rimpar, Germany) was used to maintain body temperature at + 37 °C. Scout images were obtained in coronal, axial, and sagittal planes to accurately determine the area of the rat brain to be scanned. Pre- and post-contrast T1-weighted images were acquired using a sequence with a repetition time (TR) of 600 ms, echo time (TE) of 14 ms, field of view (FOV) of 50 × 50 × 17.6 mm, and final voxel resolution of 0.15 × 0.15 × 0.8 mm. T2-weighted spin-echo sequences were obtained using a turbo spin-echo (TSE) sequence with a TR of 4000 ms, TE of 75 ms, TSE factor of 10, FOV of 45 × 45 × 21.6 mm, and final voxel resolution of 0.14 × 0.14 × 1.2 mm. The MRI data were analyzed using Inveon Research Workplace v4.1 software (Siemens Medical Solutions, Malvern, PA, USA).

### Radiosynthesis of ^18^F-FOL and ^11^C-PBR28

The ^18^F-FOL tracer was prepared according to a known procedure [25] based on the [^18^F]AlF- radiolabeling technique [30]. The radiosynthesis device was set up as previously reported [31]. The total synthesis time was 77–88 min starting from the end of the bombardment. The radiochemical purity was > 95%, and molar activity was 52 ± 22 MBq/nmol (*n* = 6). The decay-corrected radiochemical yields were 28% ± 7%.

^11^C-PBR28 synthesis was performed according to the previously published method [32]. Eight batches of ^11^C-PBR28 were produced with > 99% radiochemical purity and average molar activity of 680 MBq/nmol at the end of synthesis.

### In vivo PET/CT imaging

An Inveon Multimodality small animal PET/CT scanner (Siemens Medical Solutions, Knoxville, TN, USA) was used to perform the in vivo imaging. The spatial resolution of the PET is approximately 1.6 mm for ^18^F [33], with an axial FOV of 12.7 cm and a sagittal FOV of 10 cm.

Animals were anesthetized with a mixture of isoflurane and oxygen on a heating pad, and a cannula was placed into the tail prior to radiotracer injection. Oftagel (2.5 mg/g, Santen, Tampere, Finland) was applied before imaging to maintain the humidity of the rat’s eyes. A 10-min CT scan was performed prior to PET imaging for anatomical references and attenuation correction. The 60-min dynamic PET acquisition was started after an intravenous (i.v.) tail vein injection of ^18^F-FOL (38.9 ± 2.7 MBq, *n* = 21). In addition, 30 min dynamic ^11^C-PBR28 PET (34.1 ± 3.5 MBq, *n* = 12) was acquired 4 h prior to the ^18^F-FOL imaging.

The PET data were reconstructed using an ordered-subsets expectation-maximization 2-dimensional maximum a posteriori (OSEM2D-MAP) algorithm to yield 6 × 10, 9 × 20, 4 × 60, 4 × 180, and 2 × 300 s time frames. CT images were reconstructed using a Feldkamp-based algorithm. PET images were automatically aligned to the corresponding MRI data sets and analyzed with Carimas v2.9 (Carimas, Turku PET Centre, Turku, Finland). For visualization purposes, representative PET/CT and MRI images were captured using Inveon Research Workplace v4.1 (Siemens Medical Solutions). The images were analyzed by defining a spherical region of interest (ROI) in the hemisphere with the injection-induced lesion. This ROI was then mirrored onto the contralateral hemisphere to serve as an internal reference region. Quantitative PET image analysis was facilitated by defining an ROI on the left ventricle of the heart to determine blood radioactivity concentration. Time frames from 20 to 30 min post-injection were used for the quantitative PET image analysis. The results are expressed as standardized uptake values (SUVs) normalized for injected radioactivity dose and animal body weight as follows:

$$ \mathrm{SUV}=\mathrm{radioactivity}\ \mathrm{concentration}\ \mathrm{in}\ \mathrm{ROI}\ \Big({}_{\mathrm{MBq}/\mathrm{mL}\Big)}/\left[\mathrm{injected}\ \mathrm{radioactivity}\ \mathrm{dose}\left({}_{\mathrm{MBq}}\right)\times \mathrm{animal}\ \mathrm{weight}\left({}_{\mathrm{kg}}\right)\right] $$

and as SUV ratio = SUV _max,Lesion_/SUV_mean,Contralateral_.

### In vivo stability and modeling of ^18^F-FOL PET data

Blood samples from healthy Lewis rats (*n* = 12) were withdrawn at 2–60 min after ^18^F-FOL injection (*n* = 3 per time point) into heparinized tubes. Radioactivity concentration in whole blood and plasma was measured with a gamma counter (1480 Wizard 3″; Perkin Elmer/Wallac, Turku, Finland). Plasma proteins were separated from plasma by adding an equal volume of acetonitrile followed by centrifugation 2100×*g* for 4 min at room temperature. The plasma supernatant was then filtered through a 0.45 μm Minispike filter (Waters Corporation, Milford, MA, USA) for analysis by HPLC. A semi-preparative C18 column (Jupiter Proteo 90 Å, 4 μm, 250 × 10 mm, Phenomenex Inc., Torrance, CA, USA) was used for HPLC analysis of the plasma samples with both the ultraviolet (254 nm) and radioactivity detection. Solvent A was water containing 0.1% trifluoroacetic acid (TFA) and solvent B was acetonitrile containing 0.1% TFA. The elution was programmed as follows: 8% B during 0–1 min, from 8 to 23% B during 1–14 min, and from 23 to 8% B during 14–15 min. The flow rate was 5 mL/min. The fraction of intact tracer in the plasma was determined by comparing it with ^18^F-FOL standard.

Dynamic PET images of EAE rats were analyzed by the graphical Logan method using an image-derived input function corrected for metabolites with the above population-based information and plasma/blood ratio of radioactivity. Distribution volumes, distribution volume ratios, and brain-to-blood ratios were computed for EAE lesions and contralateral brain hemisphere ROIs.

### Ex vivo biodistribution

Following the 60 min dynamic in vivo PET imaging, the rats were sacrificed for ex vivo autoradiography and biodistribution analysis (day 14, *n* = 6; and day 90, *n* = 4) by increasing the anesthetic to a terminal level (4–5% isoflurane; and oxygen, 500–700 mL/min), and blood was then removed from the left ventricle of the heart via cardiac puncture, and the euthanasia was confirmed by cervical dislocation. The brain and other relevant tissues were dissected and weighed, and their radioactivity was measured with a gamma counter (Triathler 3′′, Hidex, Turku, Finland). The results are expressed as a percentage of the injected radioactivity dose per gram of tissue (%ID/g).

### Digital autoradiography of brain sections

The brains were collected, snap-frozen in a bath of isopentane at a temperature of − 70 °C, and sectioned (20 μm for ex vivo autoradiography and 10 μm for histology) in a microtome (Leica CM 3050 S cryostat, Leica Biosystems, Nussloch, Germany). Coronal sections were taken from brain regions (striatum and cerebellum) and cut to positively charged slides (Superfrost Ultra Plus, Thermo Fisher, Pittsburgh, PA, USA). Then, the 20 μm sections were exposed to a phosphor imaging plate (BAS-TR2025, Fuji Photo Film Co, Ltd., Tokyo, Japan) for periods of 220 min (i.e., two physical half-lives of ^18^F) or 40 min (i.e., two physical half-lives of ^11^C). The plates were then scanned with a phosphor imaging plate reader (BAS-5000, Fuji; 25 μm internal resolution) to acquire data for autoradiography. Finally, the sections were frozen at − 20 °C. The 10 μm sections were frozen immediately after sectioning and stored at − 20 °C for histology and immunohistochemistry.

The ex vivo autoradiography data were analyzed with AIDA Image analyzer v4.55 software (Raytest Isotopenmessgeräte GmbH, Straubenhardt, Germany) according to previously described methods [5] to obtain the count densities (photostimulated luminescence per square millimeter; PSL/mm^2^) of ^18^F-FOL or ^11^C-PBR28 binding within ROIs. The following calculation was performed to quantify the bound-to-free ratio of the radioligand:

$$ \left[{\left(\mathrm{PSL}/{\mathrm{mm}}^2\right)}_{\mathrm{Lesion}}-{\left(\mathrm{PSL}/{\mathrm{mm}}^2\right)}_{\mathrm{Contralateral}}\right]/{\left(\mathrm{PSL}/{\mathrm{mm}}^2\right)}_{\mathrm{Contralateral}} $$

The bound-to-free ratio was individually calculated from each brain slice and averaged across all sections to obtain an overall bound-to-free-ratio for each rat.

### In vitro ^18^F-FOL study

To evaluate the specificity of ^18^F-FOL binding in Type I CNS inflammatory lesions, 20 μm cryosections of *f*DTH-EAE rat brain were used. First, the brain cryosections were pre-incubated in phosphate-buffered saline (PBS) at room temperature for 15 min, and then with 0.2 nM ^18^F-FOL in PBS. Only ^18^F-FOL was applied for one group of slides, while another group received a 100-fold molar excess of folate glucosamine as a blocking agent (C_25_H_30_N_8_O_10_; molecular weight, 602.56) prior to applying ^18^F-FOL for 30 min. The slides were then washed with ice-cold PBS, dipped in ice-cold distilled water, dried, and further processed and analyzed as described above.

### Histology, immunohistochemistry, and immunofluorescence

Acetone-fixed or formalin-fixed 10 μm sections were stained with hematoxylin-eosin (H&E) or Luxol Fast Blue (LFB) with cresyl violet counterstain according to standard procedures. For immunohistochemistry, the sections were post-fixed with periodate-lysine-paraformaldehyde (PLP) for 20 min [34] and washed with PBS. PLP fixation was followed by antigen retrieval in a hot citrate buffer (pH 6.0) and cooling for 20 min. The desired primary antibody: (1) anti-inducible nitric oxide synthase (iNOS, 1:500 dilution, Abcam, Cambridge, UK) to study iNOS expressing macrophages/microglia, (2) anti-mannose receptor C-type 1 (MRC-1, 1:2000 dilution, Abcam, Cambridge, UK) to study MRC-1 expressing macrophages/microglia, (3) anti-CD68 (1:1000 dilution, AbD Serotec, Hercules, CA, USA) to study macrophages. or (4) anti-FR-β (1:50 dilution, m909, a kind gift from Professor Philip S. Low, Purdue University, West Lafayette, IN, USA) to study FR-β expression [35], was added for a 1 h incubation. Incubation was followed by the addition of a secondary antibody (1) for anti-iNOS, Dako EnVision anti-rabbit (Code K4003), (2) for anti-MRC-1, Dako EnVision anti-rabbit (Code K4003), (3) for anti-CD68, Dako EnVision anti-mouse (Code K4001), and (4) for anti-FR-β, Dako (Code P0397) Streptavidin/HRP, for 30 min, and 3,3-diaminobenzidine (DAB, Dako; Code K3468) was used as a chromogen. The sections were counterstained with hematoxylin and mounted with ProLong Gold antifade reagent (Life Technologies P36930).

For immunofluorescence, the sections were first fixed with ice-cold acetone for 3 min and washed with PBS. Then, the sections were incubated with primary anti-FR-β (1:50 dilution, m909) and anti-MRC-1 antibodies (1:2000 dilution, Abcam) or with anti-FR-β and anti-iNOS antibodies (1:500 dilution, Abcam) for 30 min at room temperature. Thereafter, the sections were incubated with fluorophore-labeled secondary antibodies (1:100 dilution, Alexa Fluor 488 or Alexa Fluor 594, Invitrogen, Waltham, MA, USA). The sections were mounted as described above.

The stained sections were evaluated under a light microscope, scanned using a Pannoramic 250 F scanner or Pannoramic Midi fluorescence scanner (3D Histech, Budapest, Hungary), and analyzed with Pannoramic viewer. The percentages of positive staining area for iNOS, MRC-1, CD68, and FR-β were determined on four brain sections and averaged for each rat. The amount of demyelination was determined from the LFB staining. The positive area of immunohistochemical staining and loss of LFB staining intensity were determined by using the automatic color deconvolution method of ImageJ v.1.48 software (National Institutes of Health, Bethesda, MD, USA). The lesion sizes were evaluated by defining ROIs representing the lesion areas on the H&E stained sections from each rat. The areas were determined as described above.

### Statistical analyses

All statistical analyses were performed with GraphPad Prism v5.01 software (Graph Pad Software Inc., La Jolla, CA, USA). The results are presented as mean ± standard deviation to two significant numbers. Non-parametric Kruskal-Wallis tests with Mann-Whitney post hoc tests were used to compare lesion tracer uptake between the studied groups and intragroup histological and immunohistochemistry data. Wilcoxon matched-pairs test was used to analyze longitudinal PET imaging studies. Spearman’s correlation was used to analyze associations between immunohistochemistry, histology, and PET data. Results with *P* values less than 0.05 were considered statistically significant.

## Results

### ^18^F-FOL and ^11^C-PBR28 radioligands are able to detect *f*DTH-EAE lesions, but only ^18^F-FOL can differentiate between acute and chronic lesions

**Fig. 2:**
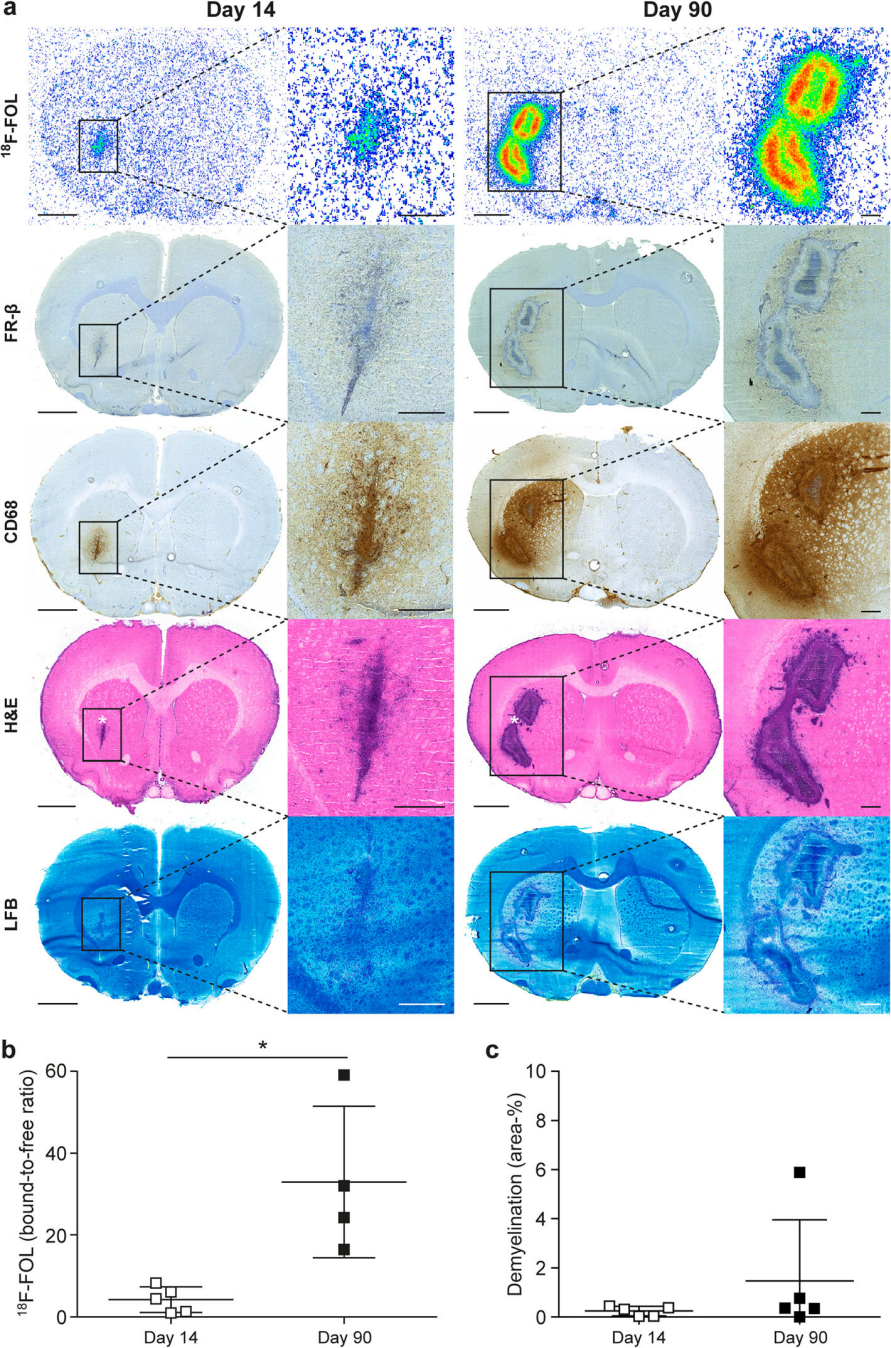
^18^F-FOL autoradiography with corresponding immunohistochemistry and histology of *f*DTH-EAE rat brains in acute and chronic phases with quantitative data. **a** Representative ex vivo ^18^F-FOL autoradiographs, anti-FR-β and anti-CD68 immunohistochemical staining, and H&E and Luxol Fast Blue (LFB) histological staining. The low-power scale bar is 2 mm, and the high-power scale bar is 50 μm. Quantification of **b** ^18^F-FOL autoradiography data and **c** demyelination from LFB staining. *P < 0.05. Error bars denote standard deviation. White asterisk in the H&E staining denotes the intracranial injection site to induce EAE

**Fig. 3:**
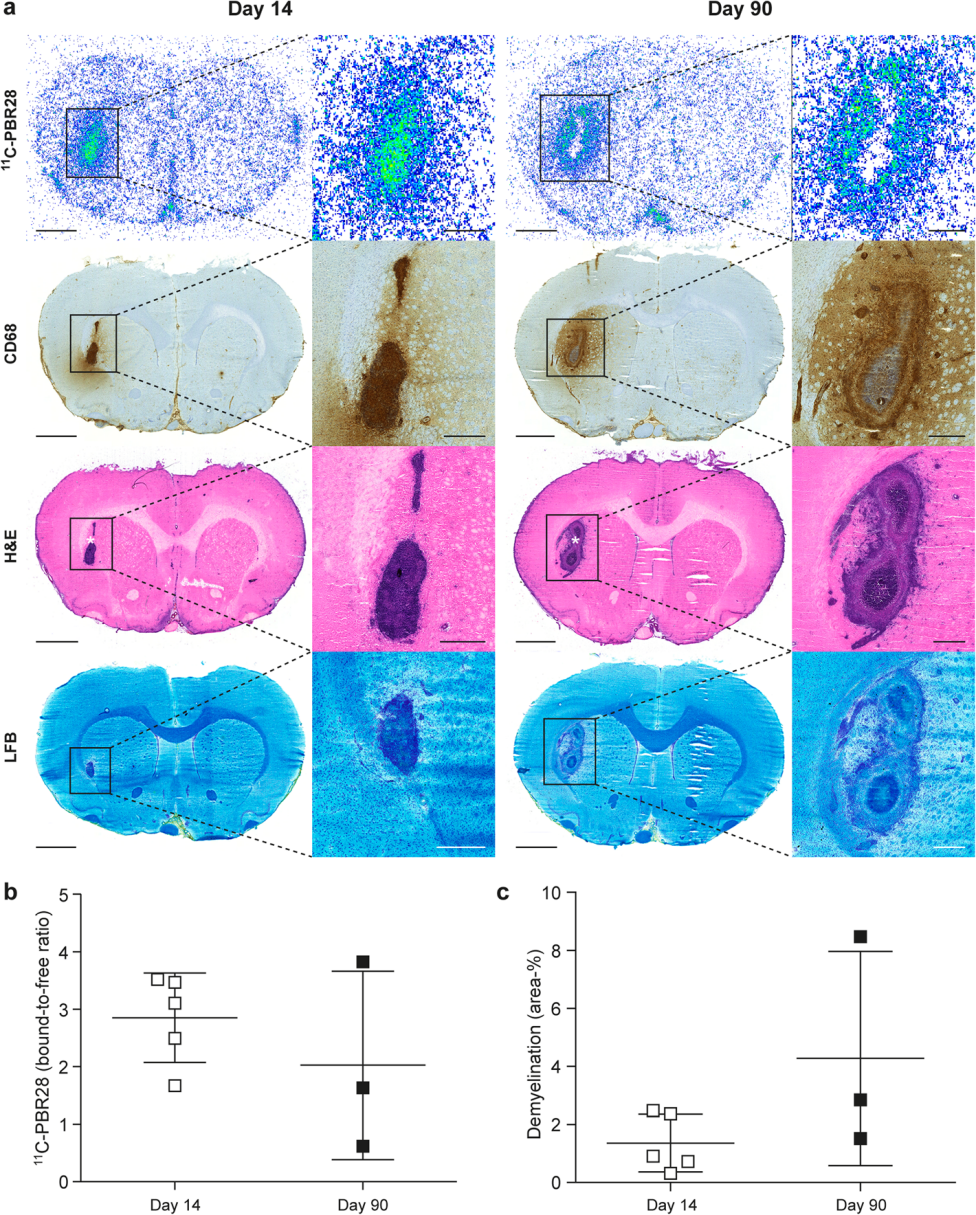
^11^C-PBR28 autoradiography with corresponding immunohistochemistry and histology of *f*DTH-EAE rat brains in acute and chronic phases with quantitative data. **a** Representative ex vivo ^11^C-PBR28 autoradiographs, anti-CD-68 immunohistochemical staining, and H&E and Luxol Fast Blue (LFB) histological staining. The low-power scale bar is 2 mm, and the high-power scale bar is 50 μm. Quantification of **b** ^11^C-PBR28 autoradiography data and **c** demyelination from LFB staining. Differences between day 14 and day 90 measures were not statistically significant (*P* > 0.05). Error bars denote standard deviation. White asterisk in the H&E staining denotes the intracranial injection site to induce EAE

**Fig. 4:**
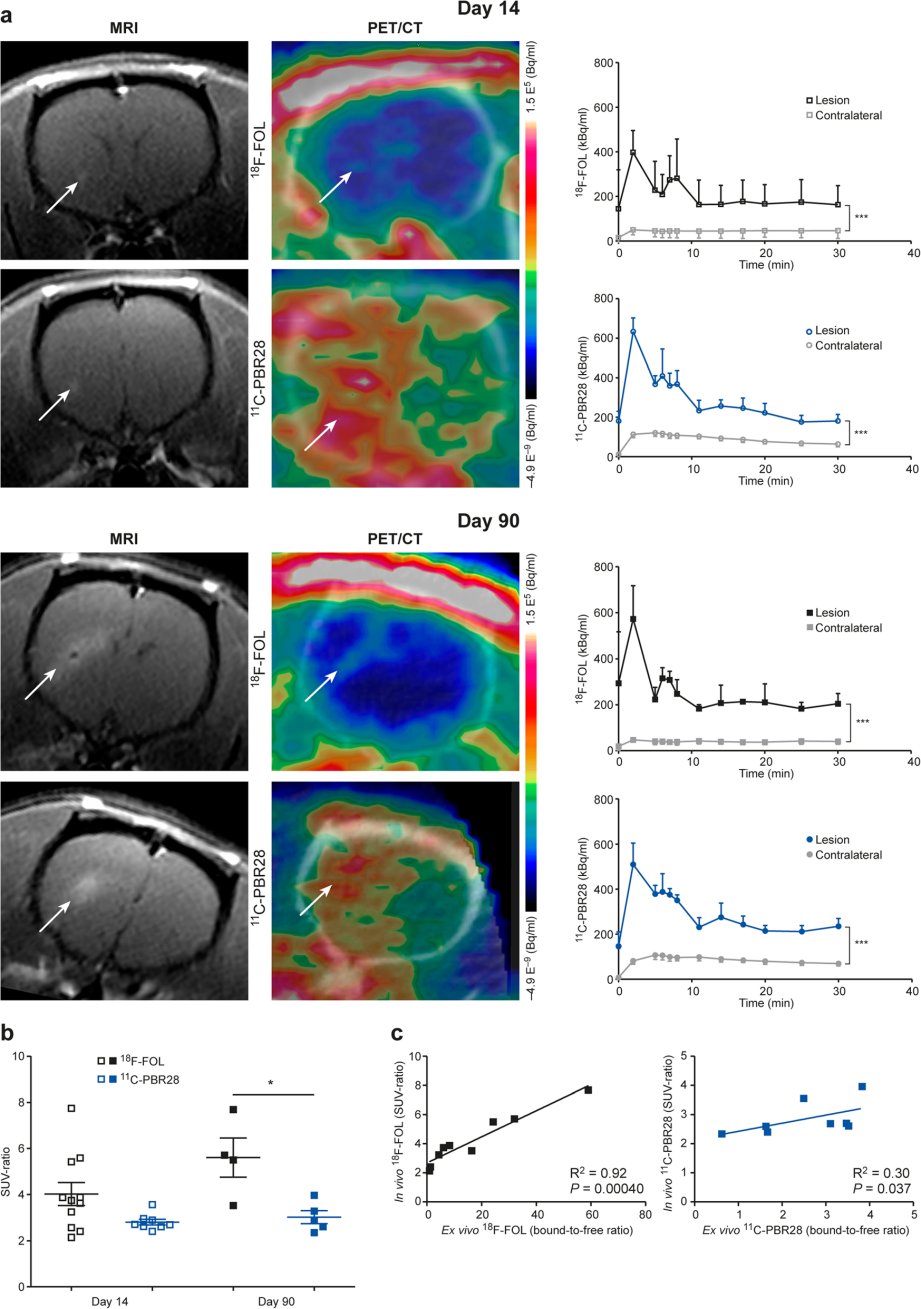
In vivo multimodal imaging of *f*DTH-EAE rat brain at acute (day 14) and chronic (day 90) phases of disease development with quantitative data. **a** Representative coronal MRI, ^18^F-FOL, and ^11^C-PBR28 PET/CT images and corresponding time-activity curves. White arrows denote inflammatory lesions. All PET images are displayed using the same color scale. Tracer uptake in the lesion is significantly higher than that in the contralateral site. ***P < 0.001. **b** Quantitative PET data presented as SUV ratios reveal significant differences between tracers in the chronic phase, but not in the acute phase. *P < 0.05. **c** Comparison of in vivo and ex vivo PET data. SUV ratio = SUV_max(lesion)_/SUV_mean(contralateral)_. *R*^2^ is Spearman’s correlation coefficient. Error bars denote standard deviation

Both ^18^F-FOL and ^11^C-PBR28 radiotracers were able to detect the inflammatory *f*DTH-EAE lesions (Figs. 2, 3, and 4). In vivo PET/CT imaging with ^18^F-FOL and ^11^C-PBR28 showed focal uptake in the induced brain hemisphere, which was co-localized with the MRI-depicted lesion. Tracer kinetics, i.e., time-activity curves (TACs), revealed a significant difference between the lesion and contralateral hemispheres in all studied groups (*P* < 0.0001, Fig. 4a). In the chronic phase, ^18^F-FOL showed significantly higher uptake than ^11^C-PBR28 (*P* = 0.016, Fig. 4a, b) at the lesion site, but no other differences between the tracers or the acute and chronic phases were observed. A significant correlation was observed between in vivo and ex vivo measurements of both ^18^F-FOL and ^11^C-PBR28 tracers (^18^F-FOL: *R* = 0.95, *P* = 0.0004, ^11^C-PBR28: *R* = 0.76, *P* = 0.037, Fig. 4c).

**Fig. 5:**
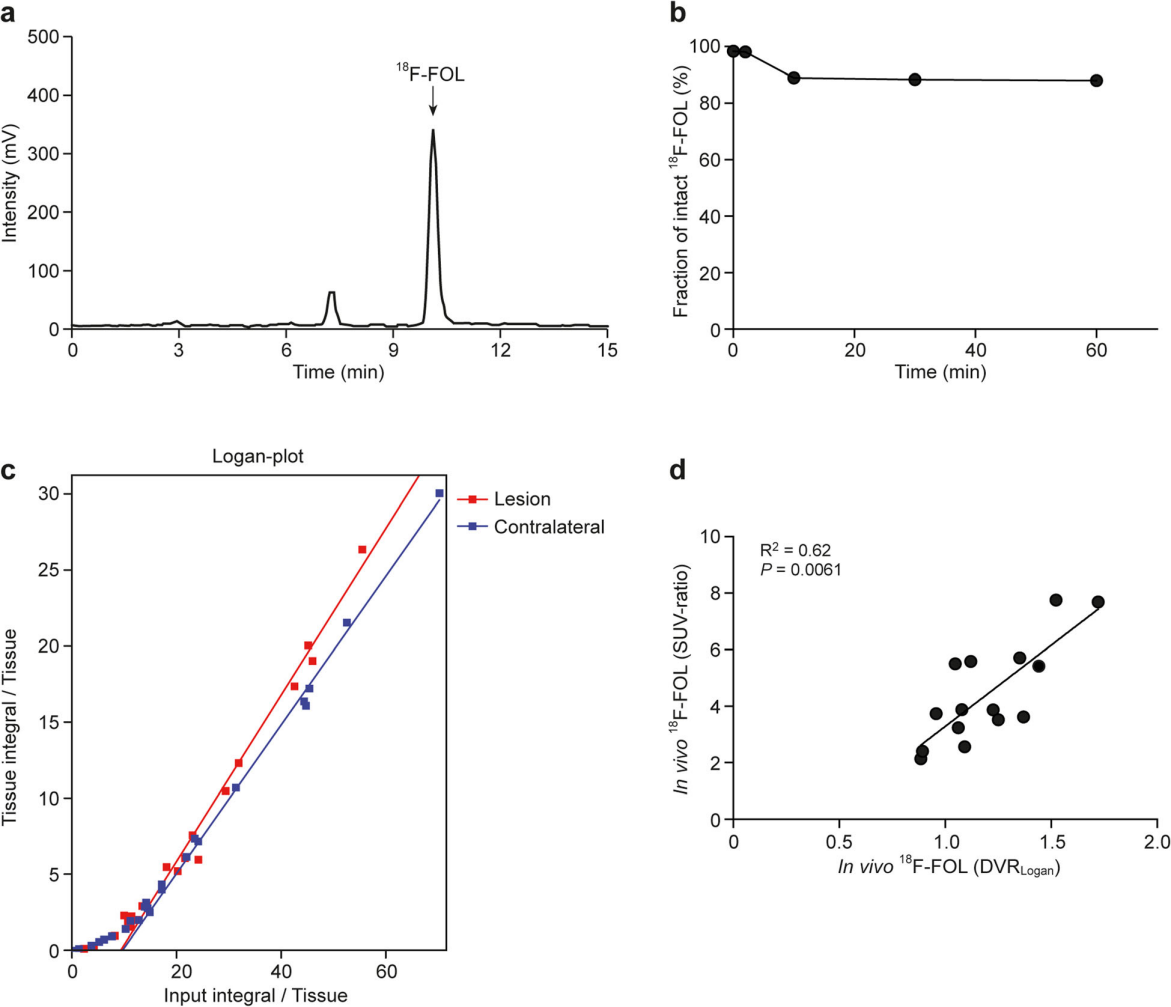
In vivo stability and modeling of ^18^F-FOL PET data. **a** Representative radio-HPLC chromatogram of rat plasma withdrawn at 60 min after ^18^F-FOL injection, **b** fraction of intact tracer as a function of time, **c** representative Logan plots, and **d** Spearman’s correlation of SUV-ratios and distribution volume ratio (DVR_Logan_)

The i.v. administered ^18^F-FOL remained very stable in rat blood circulation (88 ± 0.20% intact tracer at 60 min post-injection, Fig. 5a, b and Additional file 1: Figure S1) and the tracer uptake kinetics in the brain were well described by the reversible model (Fig. 5c). The Logan plot-based distribution volume ratio (DVR, lesion/contralateral brain, Table 2) correlated well with simplified SUV-ratio (Fig. 5d).

**Table 2:** Logan plot analysis of ^18^F-FOL uptake

	DV Lesion	DV Contralateral	DVR Lesion/contralateral	Brain-to-blood ratio Lesion/contralateral
Day 14	0.84 ± 0.69	0.72 ± 0.62	1.19 ± 0.26	1.18 ± 0.28
Day 90	0.62 ± 0.070	0.52 ± 0.16	1.28 ± 0.30	1.33 ± 0.32
*P* value day 14 vs. day 90	0.33	0.33	0.54	0.38

**Fig. 6:**
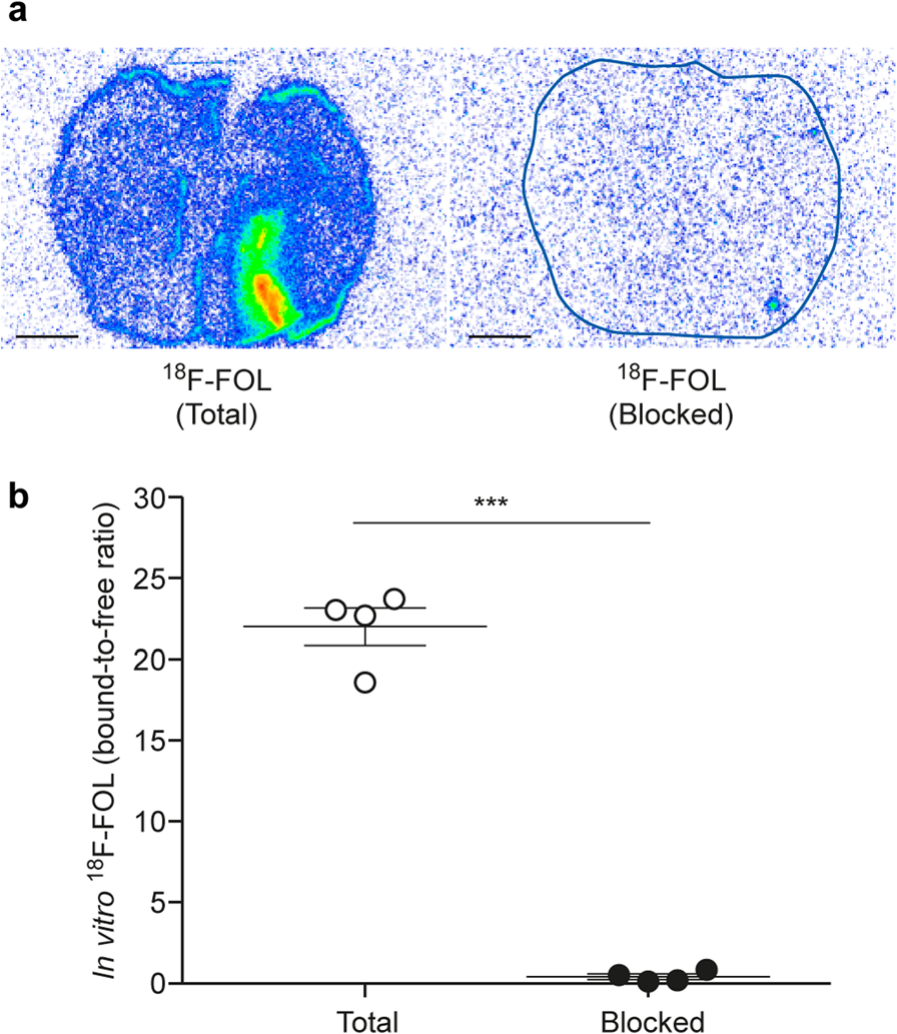
In vitro binding of ^18^F-FOL in *f*DTH-EAE rat brain cryosections. **a** Representative autoradiographs of total binding and folate glucosamine-blocked binding. The scale bar is 2 mm. **b** Quantification of ^18^F-FOL binding verifies the signal’s specificity to folate receptors (paired *t* test). Error bars denote standard deviation. ***P < 0.001

The in vitro autoradiography assay revealed significantly lower ^18^F-FOL binding to lesions from brain cryosections pre-incubated with the folate glucosamine blocking agent than in lesions not pretreated with the blocking agent, with bound-to-free ratios of 0.44 ± 0.17 vs. 22 ± 1.2, respectively (*n* = 3, *P* < 0.0001, Fig. 6a, b). This indicates that the tracer binding in lesions was specific to FRs. According to the ex vivo autoradiography, the ^18^F-FOL uptake was significantly higher during the chronic phase of *f*DTH-EAE than in the acute phase, with bound-to-free ratios of 4.2 ± 1.4 (day 14, *n* = 5) vs. 33 ± 9.3 (day 90, *n* = 4, *P* = 0.016, Fig. 2a, b). For ^11^C-PBR28, the bound-to-free ratios were 2.8 ± 0.44 (day 14, *n* = 4) vs. 2.3 ± 0.72 (day 90, *n* = 4, *P* = 0.58, Fig. 3a, b). The areas of increased ^18^F-FOL uptake were co-localized with anti-FR-β positivity, and the intensity of ^18^F-FOL binding appeared to be increased in areas around the hypercellular lesion core, where active demyelination and remyelination are known to take place in CNS lesions (Fig. 2).

**Fig. 7:**
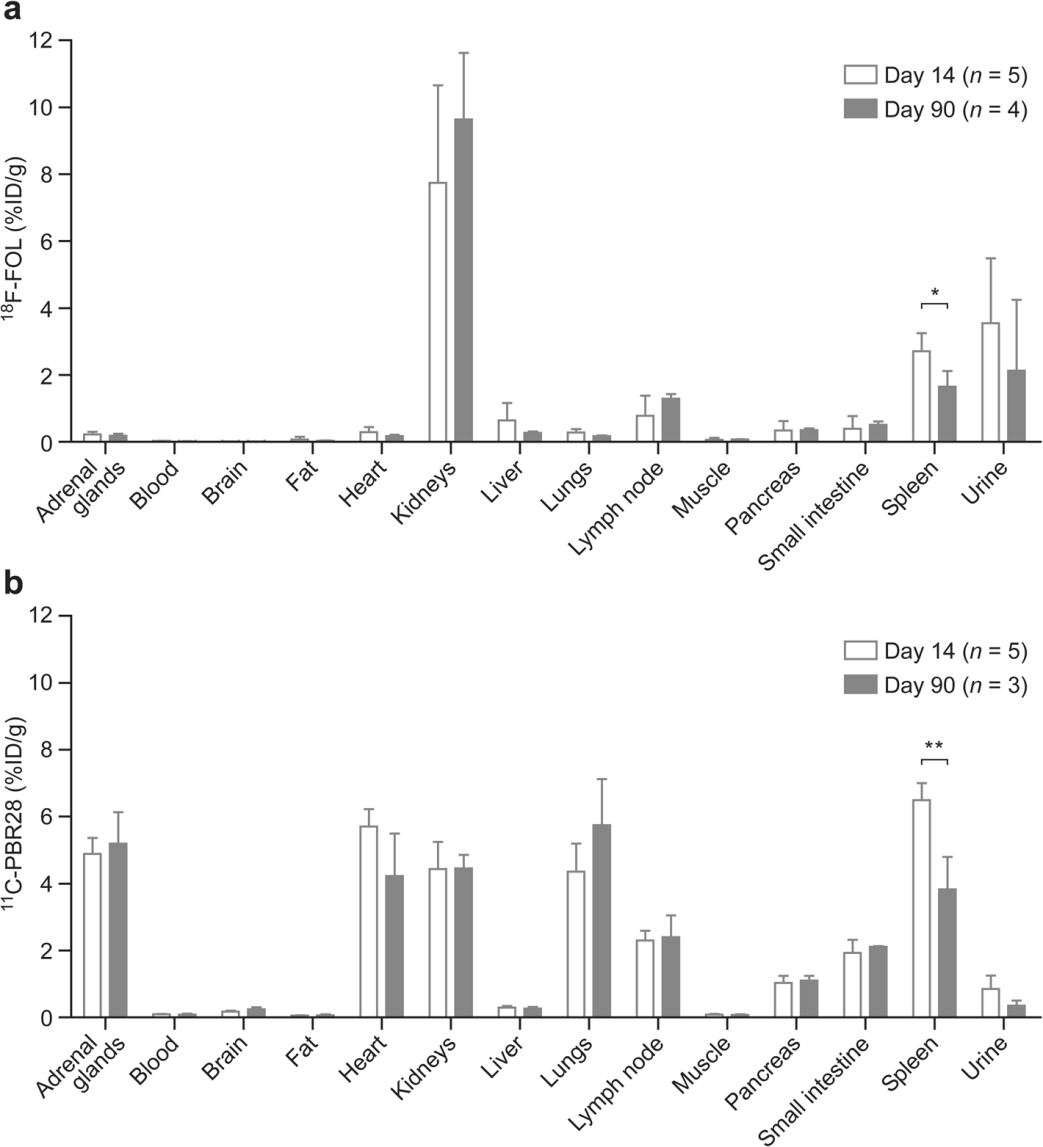
Ex vivo biodistribution of **a** ^18^F-FOL radioactivity at 60 min post-injection, and **b** ^11^C-PBR28 radioactivity at 30 min post-injection, in *f*DTH-EAE rats. *P < 0.05, **P < 0.01. Error bars denote standard deviation. Note that data from three animals are missing due to technical failure in ex vivo gamma counting

Figure 7 shows ex vivo gamma counting of the excised tissues (note, data are missing from three animals due to technical failure). The highest ^18^F-FOL uptakes were observed in kidneys, urine, and spleen. The radioactivity concentration in the spleen on day 14 was significantly higher than that on day 90 (*P* = 0.013). In the whole brain, the ^18^F-FOL uptake showed similar levels in both the acute and chronic phases of *f*DTH-EAE (*P* = 0.78). By contrast, ^11^C-PBR28 showed the highest radioactivity uptake in spleen, adrenals, heart, lungs, and kidneys. In spleen (*P* = 0.0019), the uptake was significantly higher in the acute phase than in the chronic phase.

### FR-β is expressed in acute and chronic *f*DTH-EAE lesions and is related to the anti-MRC-1 positive macrophage and microglia phenotype

**Fig. 8:**
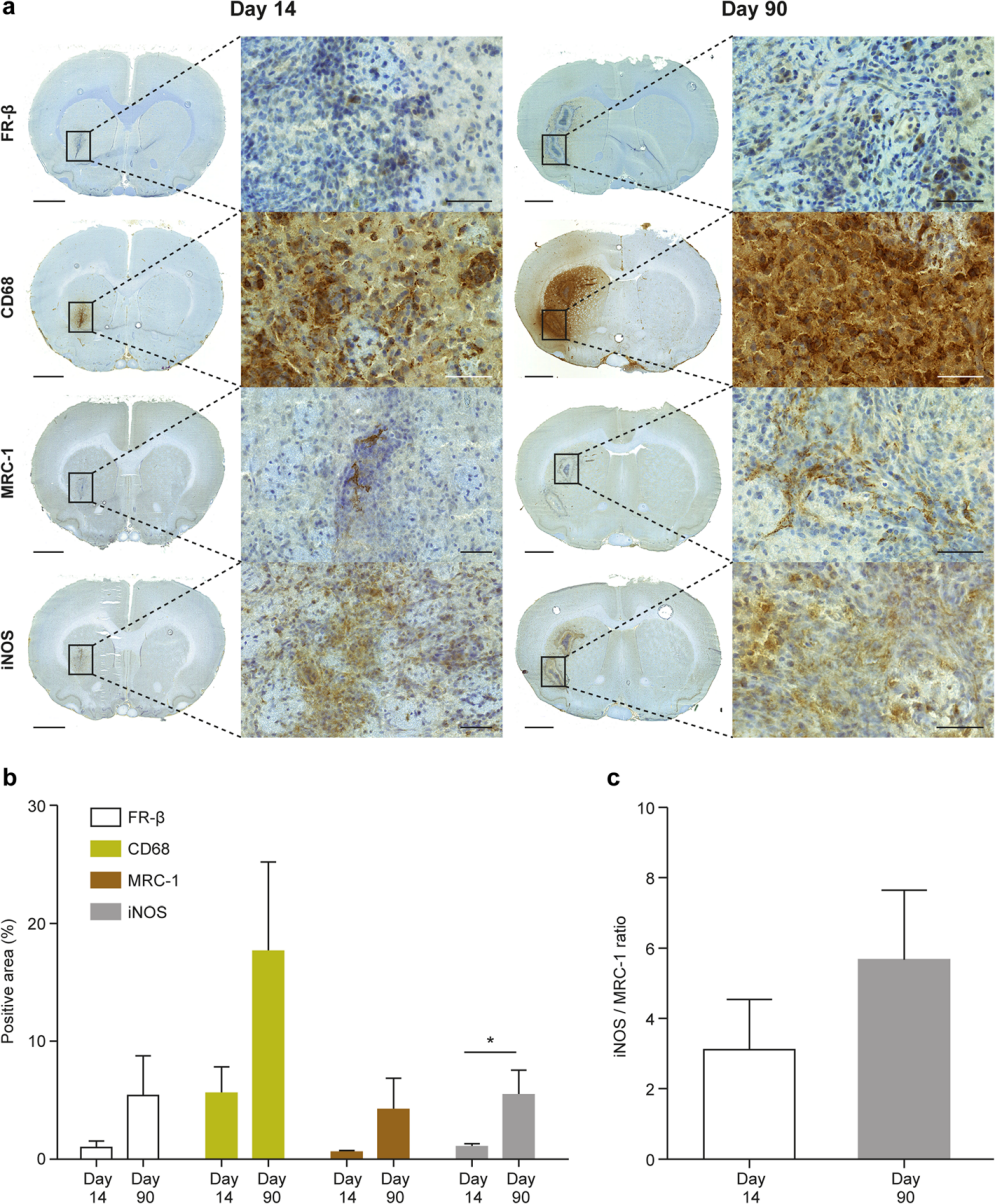
Immunohistochemical evaluation of acute (day 14) and chronic (day 90) *f*DTH-EAE rat brain lesions. **a** Representative anti-FR-β, anti-CD68, anti-MRC-1, and anti-iNOS staining. The low-power scale bar is 2 mm, and the high-power scale bar is 50 μm. **b** Quantification of immunohistochemistry data. The area of anti-iNOS positivity is significantly higher in the chronic phase than in the acute phase. The positive staining area was normalized to the lesion hemisphere area (positive area %). *P < 0.05. **c** The iNOS/MRC-1 ratio was higher in the chronic phase than in the acute phase, but the difference did not reach statistical significance (*P* > 0.05). Error bars denote standard deviation

The induction of *f*DTH-EAE in rats resulted in MS-like focal lesions with CD68 and FR-β positive cells (Fig. 8a, b). On day 14, FR-β expression was already present in the lesion site and remained prominent when the disease progressed to the chronic phase. The healthy rat showed no FR-β positive cells in the brain (Additional file 2: Figure S2). Interestingly, anti-FR-β immunohistochemistry, H&E staining, and LFB staining all revealed that FR-β positive cells were concentrated mainly in the areas outlining the lesions, with some positivity being detected in active demyelinating and remyelinating areas and in areas of NAWM (Figs. 2 and 3). The level of demyelination observed on LFB staining showed no difference between acute and chronic *f*DTH-EAE (Figs. 2a, c and 3a, c).

According to the immunohistochemical evaluations, the positive staining areas of anti-FR-β [acute phase, 1.0% ± 0.56% (*n* = 10) vs. chronic phase, 5.4% ± 3.4% (*n* = 7), *P* = 0.11] and anti-CD68 [acute phase, 5.6% ± 2.2% (*n* = 10) vs. chronic phase, 18% ± 7.5% (*n* = 7), *P* = 0.23] were higher during the chronic disease stage when normalized to the area of the lesioned hemisphere (Fig. 8a, b), but the differences did not reach statistical significance. The same was also true for the anti-MRC-1 staining positivity (acute phase, 0.61% ± 0.12% (*n* = 9) vs. chronic phase, 4.2% ± 2.6% (*n* = 7), *P* = 0.14, Fig. 8a, b). By contrast, the anti-iNOS positive area was significantly higher during the chronic phase than in the acute phase (acute phase, 1.1% ± 0.25% (*n* = 10) vs. chronic phase, 5.5 ± 2.1 (*n* = 7), *P* = 0.019, Fig. 8a, b). Accordingly, the iNOS/MRC-1 ratio was slightly higher in the chronic *f*DTH-EAE lesions (day 14: 3.2 ± 1.4 (*n* = 9) vs. 5.7 ± 2.0 (*n* = 7), *P* = 0.21, Fig. 8c) than in the acute lesions. The size of the lesions significantly increased as the disease progressed from the acute phase to the chronic phase (0.061 ± 0.027 mm^2^ vs. 1.3 ± 0.51 mm^2^, *P* = 0.012, Table 1).

**Fig. 9:**
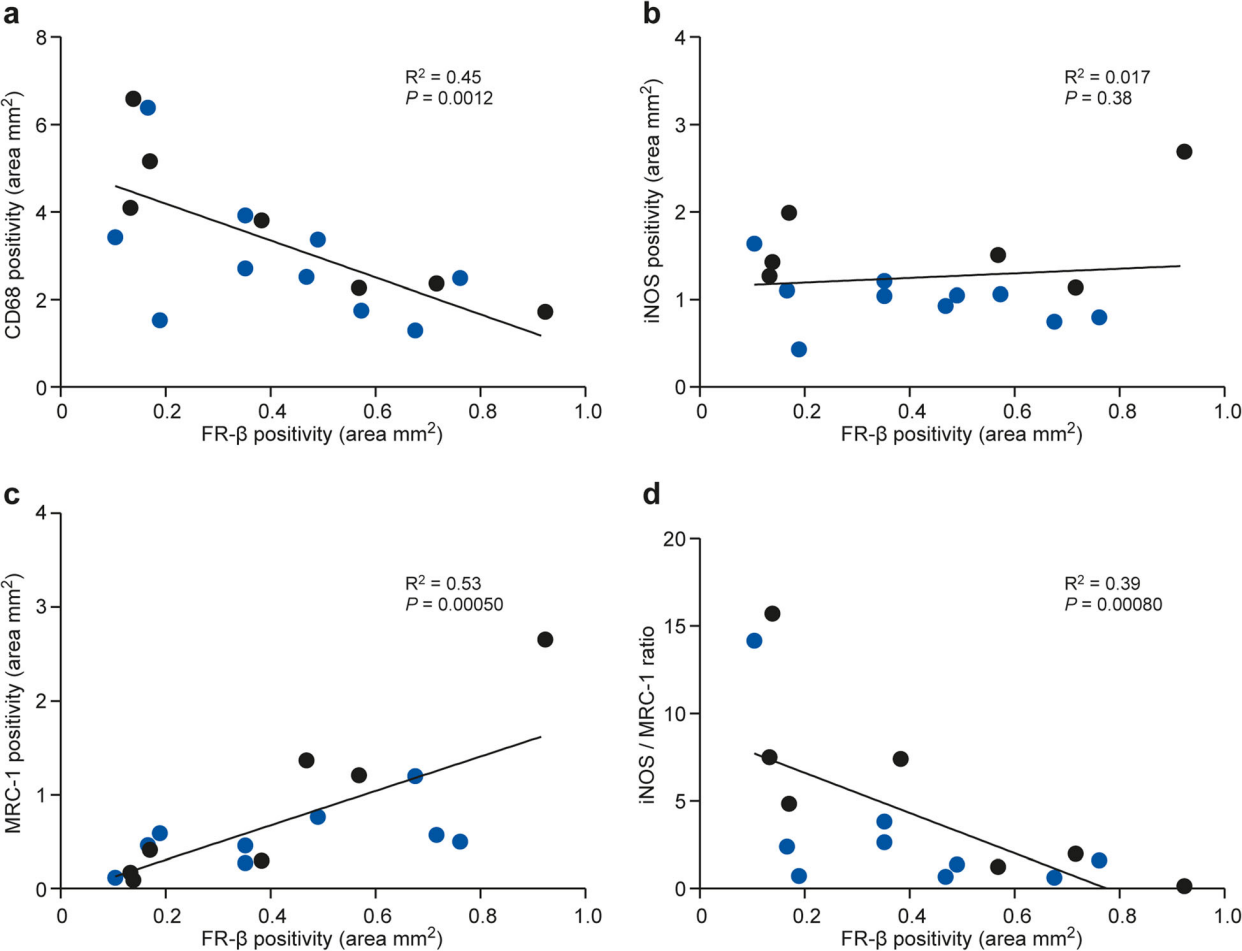
Comparison of anti-FR-β positivity and macrophage/microglia phenotype markers. There is a negative correlation between anti-FR-β positivity and **a** anti-CD68-positivity, but **b** anti-iNOS positivity showed no correlation with anti-FRβ positivity. A positive correlation was further found **c** between anti-FR-β and anti-MRC-1 positivity. By contrast, **d** the iNOS/MRC-1 ratio showed a negative correlation with anti-FR-β positivity. The values were adjusted to lesion size. Blue dots represent data points from acute phase lesions, and black dots represent data points from chronic-phase lesions. *R*^2^ is Spearman’s correlation coefficient

The percentage area with anti-FR-β positivity correlated with that of anti-CD68 (*R* = − 0.72, *P* = 0.0012, Fig. 9a) and anti-MRC-1 (*R* = 0.77, *P* = 0.00050, Fig. 9c). Anti-FR-β positivity showed a significant negative correlation with the iNOS/MRC-1 ratio (*R* = − 0.75, *P* = 0.00080, Fig. 9d), but displayed no correlation with anti-iNOS positivity (*R* = − 0.24, *P* = 0.38, Fig. 9b). The other correlations showed no significant differences between acute and chronic phases.

**Fig. 10:**
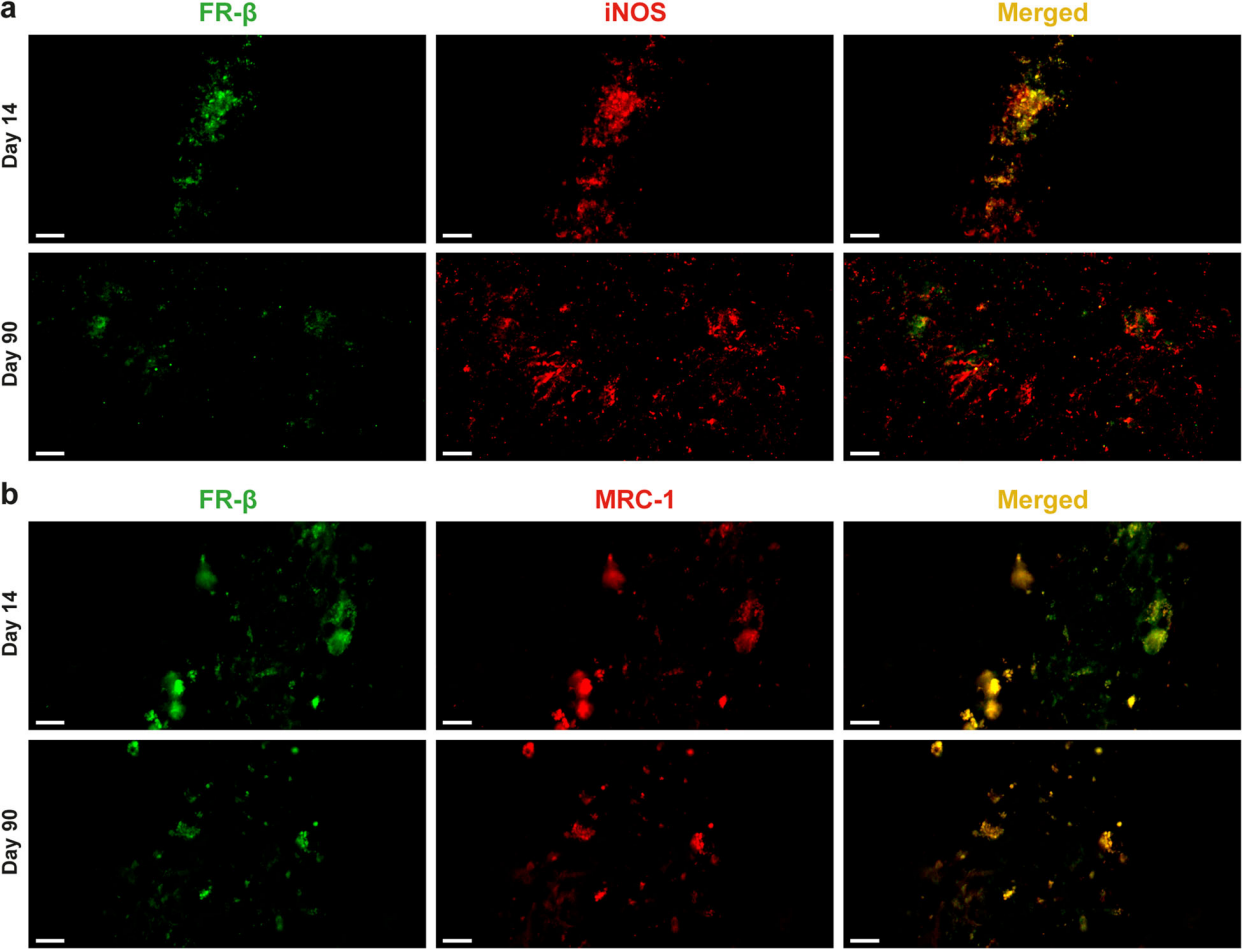
Double immunofluorescence staining for FR-β and iNOS- and MRC-1-positive macrophages/microglia of *f*DTH-EAE rat brain lesions. **a** Anti-iNOS and **b** anti-MRC-1 co-localize with anti-FR-β in both the acute (day 14) and chronic (day 90) lesions (yellow color in Merged images), but MRC-1 more prominently. High power scale bar is 20 μm

The double immunofluorescence staining further confirmed that anti-FR-β positivity in the *f*DTH-EAE lesions co-localized with both anti-iNOS and anti-MRC-1, but more prominently with anti-MRC-1 (Fig. 10a, b).

## Discussion

The role of macrophages and microglia in MS and EAE pathogenesis has been documented in several previous studies, but it has remained unclear whether activated macrophage-associated FR-β is also present in CNS inflammation. In this study, we evaluated the expression patterns of FR-β in *f*DTH-EAE inflammatory lesions using anti-FR-β immunohistochemistry and the FR-binding radioligand ^18^F-FOL. The findings are unique and reveal that FR-β is expressed during both acute and chronic type I inflammatory CNS lesions in *f*DTH-EAE rat models mimicking MS, and that ^18^F-FOL is able to visualize these lesions.

The data from this study are in line with previous research suggesting that FR-β expression is present in EAE [36]. In our *f*DTH-EAE rat model, the FR-β expression was mainly detected in areas outlining the CNS lesions. These areas typically exhibit active demyelination, active remyelination, and inflammation, all of which are known to have an important role in MS pathology [1, 9, 36]. Previously, FR-targeted aminopterin therapy was found to effectively reduce the degree of inflammation and demyelination in acute myelin basic protein (MBP)-induced EAE, resulting in improved clinical scores in rats [36]. The fact that co-administration of excess folate with the folate-aminopterin therapy abrogates any therapeutic effect confirms that the uptake of the folate-aminopterin conjugate is FR-mediated. This finding, together with our data, indicates that FR-β might also play an important role in the development of inflammatory lesions in MS.

Both ^18^F-FOL and ^11^C-PBR28 PET radioligands were able to visualize acute and chronic focal EAE inflammatory lesions. However, ^18^F-FOL was able to demonstrate differences between small acute lesions and large chronic progressive lesions, differences that ^11^C-PBR28 was unable to detect in EAE (Figs. 2 and 3). In addition, ^18^F-FOL showed a lower background signal than ^11^C-PBR28 (Figs. 2 and 3). This is especially beneficial, as current activated macrophage and microglia detecting TSPO tracers, including ^11^C-PBR28, still have relatively high background signals when used to image neuroinflammation [14, 16, 17]. Hence, ^18^F-FOL shows desirable attributes for imaging of inflammatory CNS lesions. However, because the spatial resolution and sensitivity of ex vivo digital autoradiography are much better than that of in vivo PET imaging, we consider ex vivo digital autoradiography to be the most reliable method for evaluating new molecular imaging tracers in rodent models. In vivo ^18^F-FOL PET/CT showed a moderate ability to visualize changes occurring in inflammatory activity when *f*DTH-EAE lesions progressed to the chronic phase. Unfortunately, because of the smaller size of acute lesions, their detection on in vivo PET was not as optimal as with the larger chronic lesions (Table 1). The Inveon small animal PET provides a spatial resolution of approximately 1.6 mm for ^18^F [33]. Note, for logistical reasons, we were able to perform longitudinal PET/CT imaging only for some group B animals and we were unable to mix the ^11^C-PBR28/^18^F-FOL scan order, even though that was the original plan. In addition, in this study, we chose to determine the size of the lesion by ex vivo analysis because, due to random coil failure, not all MR images were of the same high quality.

The relapse in EAE is considered to be characterized by the suppression of immunomodulating Arg-1-positive macrophages and microglia in lesion sites [9, 26]. Although some uncertainty existed initially regarding how FR-β expression reflects macrophage/microglia polarization in various inflammatory conditions, it is now generally accepted that FR-β is upregulated on both iNOS-positive and IL-10-positive macrophages, with the density of FR-β being somewhat higher on IL-10-positive than iNOS-positive macrophages [37, 38]. These studies illustrate that FR-β expression profiles can be considered heterogeneous and do not fit exactly with either of these polarization patterns. Interestingly, we found a clear positive correlation between anti-FR-β and anti-MRC-1 positivity only during the chronic phase, not in the acute phase, for *f*DTH-EAE. This was further supported by anti-FR-β and anti-MRC-1 double immunofluorescence staining. If FR-β and MRC-1 expression levels are linked in rats with chronic progressive EAE, the observed correlation illustrates that MRC-1 expressing macrophages/microglia express more of FR-β than iNOS expressing macrophages/microglia.

In addition to the correlation between anti-FR-β positivity and anti-MRC-1 positivity, the data here suggest that increased anti-FR-β positivity in chronic lesions correlates with the reduced iNOS/MRC-1 ratio that is known to be associated with reduced relapse rate and spontaneous recovery in EAE rats [26]. On the basis of this observation, one can speculate that FR-β expression levels, and hence FR-β-targeted molecular probes, might have the potential to be used as surrogate markers to provide information on activated macrophage/microglia polarization patterns, and therefore aid in predicting inflammation severity and lesion progression in chronic CNS inflammatory lesions. Despite the positive correlation between anti-FR-β and anti-MRC-1 immunohistochemistry, only the anti-iNOS positivity was significantly higher in chronic than in acute focal DTH lesions. The predominant iNOS-positivity of macrophages/microglia in chronic lesions reported above seems logical, as macrophages with this polarization would be required to promote pro-inflammatory reactions. Whether an imbalance in iNOS/MRC-1 ratio towards iNOS-positive microglia is necessary for the development of chronic *f*DTH-EAE, or whether this imbalance is a consequence of chronic lesion formation, remains to be further studied.

The *f*DTH-EAE rat model has previously been used to demonstrate the diagnostic capability of a new molecular imaging method [39]. It is known that DTH lesions have the ability to progress to a chronic phase mimicking the progressive form of MS. Additionally, the *f*DTH-EAE model can be used to monitor individual lesions without disturbance from other additional lesions. Another myelin oligodendrocyte glycoprotein-induced EAE (*f*MOG-EAE) also forms individual focal lesions, but these do not develop into a chronic form, thereby limiting its value for evaluating new neuroinflammation imaging tracers. Because of these facts, the *f*DTH-EAE model was chosen for this study. However, the intracranial injection itself may cause inflammation and affect also the contralateral side of the brain. Therefore, it can be regarded as a study limitation that healthy intact rats were not included in the study.

We previously observed that positive anti-FR-β immunohistochemistry in inflamed atherosclerotic lesions co-localizes with ^18^F-FOL binding, and that ^18^F-FOL clearly binds more to MRC-1-positive macrophages than to iNOS-positive macrophages [25]. Our present findings in MS-like inflammatory lesions of *f*DTH-EAE rat brain further corroborate the visual co-localization of anti-FR-β positivity with ^18^F-FOL binding (Fig. 2). However, although the quantification of ^18^F-FOL uptake (Fig. 2b) showed a statistically significant difference between acute and chronic *f*DTH-EAE inflammatory lesions, the difference in anti-FR-β positivity (Fig. 8b) was not significant. It is noteworthy that ^18^F-FOL is known to also bind to another isoform of FR, FR-α, [23] which occurs at very low levels in normal brain tissue in places such as the choroid plexus [40]. The observed difference between ^18^F-FOL binding and anti-FR-β positivity may possibly be due to the binding of ^18^F-FOL to FR-α in the brain, but this cannot be confirmed by the anti-FR-β immunohistochemistry. In addition, we identified that anti-MRC-1 positivity is concentrated in the regions with the highest ^18^F-FOL uptake, indicating that ^18^F-FOL binds prominently to MRC-1-positive macrophages and microglia. Indeed, this phenotype is known to be related to tissue remodeling and remyelination [9, 37]. Previously, we and others have shown that in in vitro polarized macrophages (from peripheral blood mononuclear cells), expression of FR-β is significantly increased in MRC-1 expressing macrophages, compared with iNOS-expressing macrophages [25, 41]. Whether the FR-β expression supports immunoregulatory functions, tissue remodeling, and remyelination required to recovery from chronic CNS inflammation, needs to be further investigated.

## Conclusions

Our results indicate that FR-β is expressed in activated macrophages/microglia in focal EAE lesions during both the acute and chronic phases of the disease. Folate-based PET imaging with ^18^F-FOL enables monitoring of lesion development, complementing the information that can be acquired with TSPO-targeted PET imaging. FR-β may be a useful target for both in vivo imaging and the development of new therapeutics for patients with MS.

## Supplementary information

**Additional file 1: Figure S1.** Radio-HPLC chromatogram for ^18^F-FOL standard.

**Additional file 2: Figure S2.** Representative anti-FR-β immunohistochemical staining of a healthy Lewis rat brain. There are no anti-FR-β positive cells. Low power scale bar is 2 mm and high power scale bar is 50 μm. High-power image is from the same site as the EAE-inducing injection.

## Data Availability

Data supporting the conclusions of this article are presented in the manuscript.

## Abbreviations

%ID/g: Percentage of injected radioactivity dose per gram of tissue
^11^C-PBR28: *N*-acetyl-*N*-(2-[^11^C]methoxybenzyl)-2-phenoxy-5-pyridinamine
^18^F-FOL: Aluminum [^18^F]fluoride-labeled 1,4,7-triazacyclononane-*1,4,7*-triacetic acid conjugated folate
BBB: Blood-brain barrier
BCG: Bacillus Calmette-Guérin
CD68: Cluster of differentiation 68
CFA: Complete Freund’s adjuvant
CNS: Central nervous system
CT: Computed tomography
EAE: Experimental autoimmune encephalomyelitis
*f*DTH-EAE: Focal delayed-type hypersensitivity model of experimental autoimmune encephalomyelitis
FOV: Field of view
FR: Folate receptor
FR-α: Folate receptor-α
FR-β: Folate receptor-β
Gd: Gadolinium
H&E: Hematoxylin-eosin
HPLC: High-performance liquid chromatography
i.v.: Intravenous(ly)
iNOS: Inducible nitric oxide synthase
LFB: Luxol Fast Blue
MBP: Myelin basic protein
MRC-1: Mannose receptor C-type 1
MRI: Magnetic resonance imaging
MS: Multiple sclerosis
NAWM: Normal appearing white matter
OSEM2D-MAP: Ordered-subsets expectation maximization 2-dimensional maximum a posteriori
PBS: Phosphate-buffered saline
PET: Positron emission tomography
PLP: Periodate-lysine-paraformaldehyde
PSL/mm^2^: Photostimulated luminescence per square millimeter
ROI: Region of interest
RRMS: Relapsing-remitting multiple sclerosis
s.c.: Subcutaneous(ly)
SPE: Solid-phase extraction
SUV: Standardized uptake value
TAC: Time-activity curve
TB: *Mycobacterium tuberculosis*
TE: Echo time
TR: Time of repetition
TSE: Turbo spin-echo
TSPO: Translocator protein 18 kDa
