# Supplementary figures and images for: Folate receptor-targeted positron emission tomography of experimental autoimmune encephalomyelitis in rats
Source: J Neuroinflammation. 2019 Dec 3;16:252. doi: 10.1186/s12974-019-1612-3 (PMC6892159; doi:10.1186/s12974-019-1612-3)

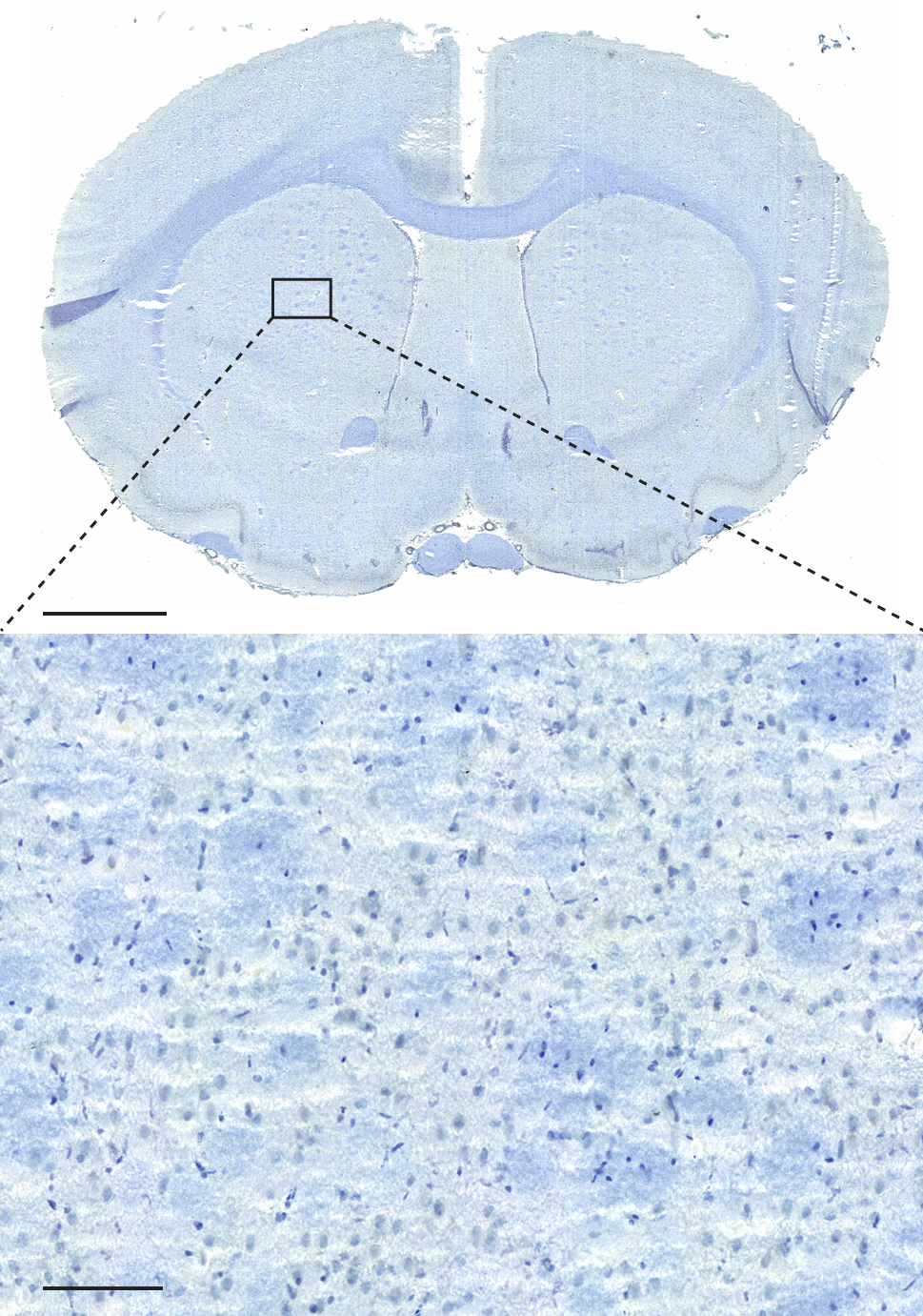

Supplement: Supplementary file 2 — Additional file 2: Figure S2. Representative anti-FR-β immunohistochemical staining of a healthy Lewis rat brain. There are no anti-FR-β positive cells. Low power scale bar is 2 mm and high power scale bar is 50 μm. High-power image is from the same site as the EAE-inducing injection. [file 12974_2019_1612_MOESM2_ESM.tif]
